# Supplementary material for: Temperature-Dependent Circularly Polarized Luminescence of a Cholesteric Copolymer Doped with a Europium Complex
Source: Polymers (Basel). 2023 Mar 8;15(6):1344. doi: 10.3390/polym15061344 (PMC10056765; doi:10.3390/polym15061344)
Supplement: Supplementary file 1 [file polymers-15-01344-s001.zip › polymers-2245739-supplementary.pdf]

Supporting information

Temperature-dependent circularly polarized luminescence of cholesteric copolymer doped with europium complex

Alexey Bobrovsky, Alexey Piryazev, Dimitri Ivanov, Makarii Kozlov, Valentina Utochnikova

a)

b)

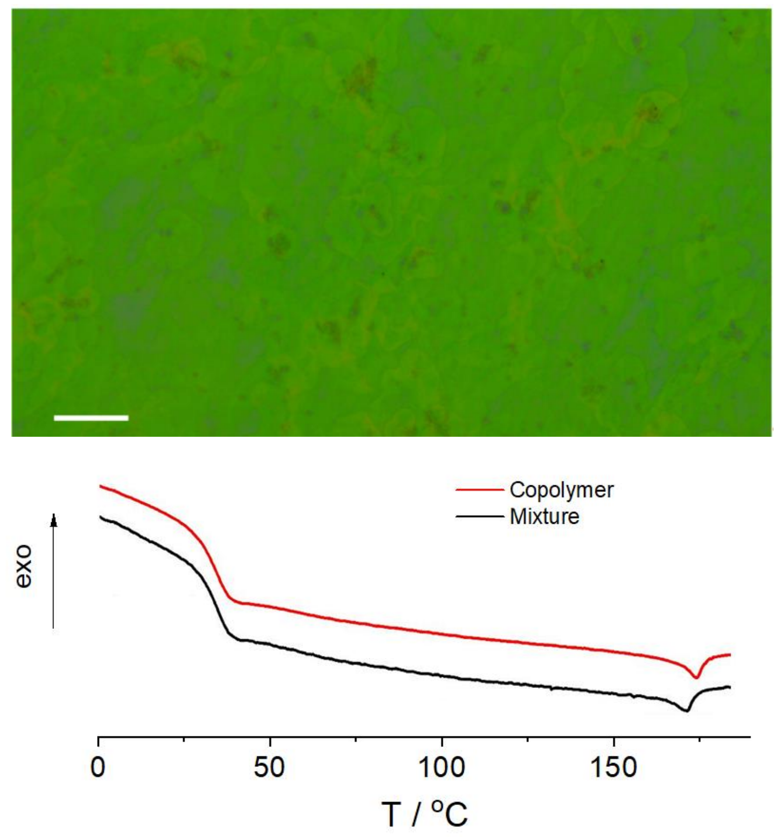

Figure S1. (a) Polarizing optical image of planarly-oriented sample of the mixture. Scale 50 µm. (b) DSC curves of copolymer and mixture; second heating scan, 10 ^{o}/min.

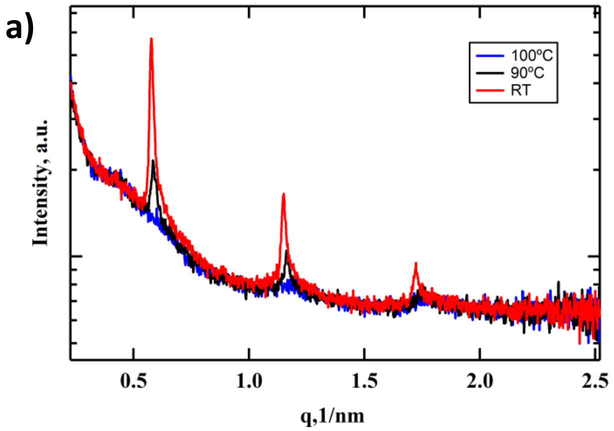

Figure S2. X-ray diffraction pattern at different temperatures (a) and temperature dependence of first diffraction order intensity (b) for copolymer.

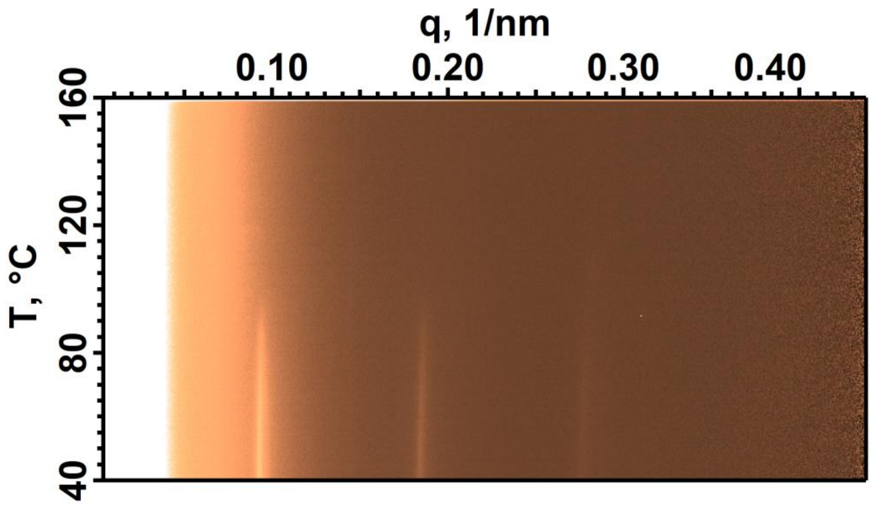

Figure S3. 2D image of temperature dependencies of X-ray diffraction for the mixture measured under cooling (1°/min).

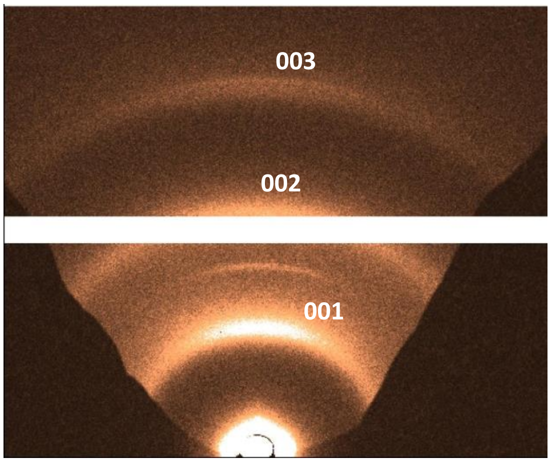

Figure S4. X-rays diffraction pattern of the copolymer fiber drawn and ca. 100 ^{o}C. Stretching direction is vertical.
